# Is It Possible to Solve the Conflicts Over Conflict of Interest? Comment on "Towards Preventing and Managing Conflict of Interest in Nutrition Policy? An Analysis of Submissions to a Consultation on a Draft WHO Tool"

**DOI:** 10.34172/ijhpm.2021.178

**Published:** 2021-12-31

**Authors:** Stella Aguinaga Bialous

**Affiliations:** Department of Social and Behavioural Sciences, School of Nursing, University of California San Francisco, San Francisco, CA, USA.

**Keywords:** Conflict of Interest, Tobacco, Food, Commercial Determinants of Health, Whole of Government

## Abstract

Addressing conflicts of interest (COIs) when developing and implementing policies to address commercial determinants of health is pivotal to ensure that these policies are free from commercial and other vested interests of unhealthy commodities industry. As a concept, this is well accepted within the tobacco control community, and supported by the existence of an international treaty, the WHO Framework Convention on Tobacco Control (FCTC). But in nutrition policy the engagement of the food industry appears to remain controversial, as efforts to create partnerships are still underway. There is a need to undertake evaluation of existing COI policies to assess their implementation and outcomes, creating best practice models that can be replicated, and understanding how to change norms within governments. Additionally, a review of existing norms, codes of conduct, and ethics to determine their impact on preventing COI would guide future implementation of these measures. Finally, governments, academics, and advocates should consider how existing tools, guidelines or other instruments could help frame the COI discussion to ensure its political feasibility. There is a need for a discussion on whether the current approach of separate policies for distinct industries is preferable than a broader COI policy that would be applicable to a wide range of unhealthy commodities and across governmental sectors.

Conflicts of interest (COIs) are, simply, when what one party wants conflicts with the wants of another party, be that an individual, group, organization or government. As Ralston and colleagues^1^ describe, however, the definition of COI varies depending on who is interpreting or framing COI. These different interpretations surfaced in the comments of the World Health Organization’s (WHO’s) tool to prevent COI in nutrition policy (WHO tool), discussed by the authors, despite the tool itself making a clear attempt to define COI and the circumstances that could lead to real or perceived conflicts. Notably, the WHO tool appears to follow the broader guidance established by the WHO’s Framework for Engagement with Non-state Actors, which requires risk assessment and due diligence for such engagements, focusing on management of conflicts that might present themselves.^2^ Indeed, Ralston et al highlight in their analysis that the public comments included differing perceptions on whether COI are to be avoided or managed.

Managing COI on the assumption that a risk management strategy is in place, and an accountability mechanism is deployed, might actually be an unrealistic expectation, especially when placed on offices who may be understaffed, overworked and assigned multiple other responsibilities. Presumably, in such cases, potential COI would have been stopped during the risk benefit assessment, but the comments described by Ralston et al confirm that finding common ground to address COI in public-private partnerships remain a complex issue at a time where such partnerships continue to grow, often to the detriment of health and development agendas.^3,4^ The underlying issue is whether a private sector actor, such as the food industry, with a fiduciary duty to deliver profits by selling unhealthy, even harmful, products, can be a partner in policies that ultimately aim at reducing the sales of these products. The same can be said for sellers of alcohol, sugared beverages, tobacco, and other unhealthy commodities.^5,6^ Irrespective of what any corporate entity may say, many commercial entities function to secure returns on investment through a model to stimulate unhealthy behaviors – and so the desire to engage in health policy counteracting that prerogative is an inherent COI. There is now sufficient evidence demonstrating that in these public-private engagements, industry’s financial bottom line is either sustained or improved.

Adding complexity to the issue is the economic power of these companies and their ability to quickly mobilize funds to alleviate communities’ suffering, especially in times of crises. There are several examples of corporate largesse during the coronavirus disease 2019 (COVID-19) pandemic, and indications of the marketing and potential policy gains from these donations.^7^ Policy-makers, particularly in low- and middle-income countries would be hard pressed to reject help in a crisis, unless they were protected by an existing whole of government approach that would guide government officials and policy-makers’ responses to private sector’s outreach efforts. It is important to note that donations, both in times of crisis and as part of “Corporate Social Responsibility” or “Sustainability” efforts, whether overtly intended or otherwise, impact public interests as they may erode existing regulations and prevent additional regulations from being imposed. A multi-sectoral guidance on engaging with private interests, combined with regulations that aim to achieve prevailing health interests (for example, on marketing), could curtail the real or perceived COI embedded in these donations. Additionally, resource-constrained countries could implement taxation or other fees to raise funds for government programs and priorities, in times of crisis or otherwise, instead of relying on donations (raising taxes or fees during such times and for such purposes being historically a common practice). Marketing restrictions and taxation are regularly opposed by these companies, which prefer their voluntary, self-managed, marketing-driven contributions to regulatory measures.^7^

Ralston and colleagues mention the Framework Convention on Tobacco Control (FCTC), particularly Article 5.3 and its implementation guidelines,^8^ as a potential model to address COI. Unlike Article 5.3 Guidelines, the WHO tool allows for situations in which a collaboration might be deemed appropriate. The authors highlight that those opposing the WHO tool rejected using tobacco as a model, arguing that tobacco is unique amongst unhealthy commodities. However, the denormalization of the tobacco industry is the result of decades of scientific research, evidence from court cases, in addition to public health campaigns and policies that have initiated a movement to frame the tobacco industry as incompatible with health, human rights and socio-economic development. The tobacco denormalization movement is not yet completed. Article 5.3 is one of the least implemented articles of the FCTC, the tobacco industry remains a threat to achieving the treaty’s goals, and the existence of a United Nations’ convention has not stopped the tobacco industry from claiming that it should be a partner and a stakeholder on decision making on tobacco control policies.^9^ However, lessons learned from tobacco control can and must be applied when addressing other commercial determinants of health, including the food industry. While it is true that, unlike tobacco, food is essential for life, processed food specifically is not. Thus, denormalization of the food industry will likely take into account a broader range of products and goods, as well as careful definition of what constitutes the “food industry.”

There is ample evidence that the “tobacco playbook,” ie, the strategies used by the tobacco industry to block, weaken or delay health policies, are emulated by other unhealthy commodities industries.^10^ Thus, policies that attempt to prevent tobacco industry interference and COI in public policy can and should be used as models for managing COI in other industries, even while best practice implementation of these strategies are still emerging. For example, creating transparency in all meetings between government and industry either through public agenda and minutes and or through public hearings, limiting these meetings to the discussion of regulatory measures, ban industry participation from committees deliberating health policy.^11,12^ The experience from the FCTC helps identify areas where implementation of the tool could be complex even when all safeguards are properly applied.

 COI policies that are narrowly focused on one health concern or industry, initiated, and mostly applied by a Ministry of Health, and without a multi-sectoral approach or support, may allow for commercial influence in policy making through ministries of Finance, Agriculture, Trade, and even within the health sector. Commercial entities exert political and economic influence through a whole of government approach and should be countered in an equivalent manner. Similarly, codes of conduct or ethics that are narrowly applied to one health issue and may not always align with existing measures on anti-corruption, ethics and transparency in a broader scale pose the risk of being ignored by policy-makers. In some countries, existing norms on COI could be leveraged and applied to nutrition policy, and to addressing COI with private entities. While this application would still require a concerted effort to ensure both awareness and applicability of these existing norms, it has the benefit of building on existing expectations and definitions of COI. Further, these are often applicable to a range of governmental sectors if not comprehensively all public sectors, hence having an advantage to potentially close loopholes otherwise prone to corporate exploitation. This might be an idealized scenario, as even countries with well-established and robust codes of conducts can have different interpretations of conflict, different understandings for the appropriateness of public-private partnerships, or weak incentives for compliance or weak mechanisms for enforcement.

## The Way Forward

 There is reason for optimism. Instruments and guidelines exist, and the past two decades have yielded significant discussions on trade and health, rules of engagement with non-State actors, the acceptance of commercial determinants of health as both an area of research as well as advocacy, which all indicate that progress is occurring. As one example, the level of awareness about the role of the tobacco industry in causing the tobacco-related pandemic has radically increased. That progress and how it came about can help leapfrog progress in addressing COI in dealing with other commercial entities and prevent treating COI in public policy as a subjective concept.

Several strategies could support the acceleration of addressing COI within commercial determinants of health to prevent interference in the public policy process. First, it is necessary to evaluate implementation and outcomes in the few countries where any measure to prevent, or manage, COI have been attempted. For example, thirteen years after approval of the guidelines to implement Article 5.3, there is a paucity of evidence on what components of it were successful, and why. Changing the norms of government behavior is a complex undertaking, and we need evidence on the best approaches to successfully promote, and enforce,^6,13^ these changes.

 Second, a review of existing norms, codes of conduct, and ethics, along with an assessment of their impact on preventing COI, would yield success stories ensuring that these efforts are accepted, implemented, and produced the desired outcomes.

Third, governments, academics, and advocates should consider how existing tools, guidelines or other instruments could help frame the COI discussion. Those engaged in addressing commercial determinants of health should ensure that their network includes human rights expertise and framing. While the WHO tool discussed by Ralston and colleagues was partly informed by human rights concepts, in the proposed implementation and comments it was unclear that a human rights framing supported the tool. Research is necessary to understand how the United Nations’ guidance on business and human rights^14^ and the guidelines on cooperation with business sector,^15^ while arguably business-friendly, could be leveraged in the evaluation and framing of COI. For example, the United Nations guidance on businesses’ respect of human rights might be contradictory to unhealthy commodities industry which, by the nature of its products, infringe on right to health. Further, it is important to understand if these, and other, documents are being used, if they are effective, and if they support a human rights approach to address commercial determinants of health.

 Fourth, we must counter commercial interests’ highjack of the Sustainable Development Goals (SDGs), including Goal 17. There is nothing in Goal 17 that would support unchecked partnerships with the private sector, especially partnerships that could harm achieving any other Goal. While it is possible that there are some models of corporate engagement with SDG, the unhealthy commodities’ industry appears to be reframing their corporate responsibility efforts as contributions to SDG without significantly changing their corporate practices and behavior.

 All this evidence could indicate the way forward to a comprehensive approach for addressing COI with the commercial sector in health policy. A whole of government approach to preventing COI in health policy might be a challenging path, but there are several points of progress suggesting it is achievable. A singular focus on a coherent strategy could lead to the development of policies addressing commercial determinants of health that are free of commercial and other vested interests, respect human rights, and promote social and economic development.

## Ethical issues

 Not applicable.

## Competing interests

 Author declares that she has worked with the WHO and Secretariat of the WHO FCTC in the implementation of Article 5.3 Guidelines.

## Author’s contribution

 SAB is the single author of the paper.

## References

[1] Ralston R, Hil SE, da Silva Gomes F, Collin J (2021). Towards preventing and managing conflict of interest in nutrition policy? an analysis of submissions to a consultation on a draft WHO tool. Int J Health Policy Manag.

[2] World Health Organization. Framework for Engagement with Non-state Actors. WHO. https://www.who.int/about/collaborations/non-state-actors/A69_R10-FENSA-en.pdf.

[3] Ruckert A, Labonté R (2014). Public–private partnerships (ppps) in global health: the good, the bad and the ugly. Third World Q.

[4] Hernandez-Aguado I, Zaragoza GA (2016). Support of public-private partnerships in health promotion and conflicts of interest. BMJ Open.

[5] Collin J (2021). Taking steps toward coherent global governance of alcohol: the challenge and opportunity of managing conflict of interest. J Stud Alcohol Drugs.

[6] Swinburn BA, Kraak VI, Allender S (2019). The global syndemic of obesity, undernutrition, and climate change: the Lancet Commission report. Lancet.

[7] Collin J, Ralston R, Hill S, Westerman L. Signalling Virtue, Promoting Harm: Unhealthy commodity industries and COVID-19. 2020. https://ncdalliance.org/sites/default/files/resource_files/Signalling%20Virtue%2C%20Promoting%20Harm_Sept2020_FINALv.pdf. Accessed June 14, 2021.

[8] Conference of the Parties (COP) of the WHO Framework Convention on Tobacco Control. Guidelines on the protection of public health policies with respect to tobacco control from commercial and other vested interests. (COP Decision FCTC/COP3(7). https://www.who.int/fctc/guidelines/article_5_3.pdf. Accessed December 9, 2018. Published November 2008.

[9] Bialous SA (2019). Impact of implementation of the WHO FCTC on the tobacco industry’s behaviour. Tob Control.

[10] Mialon M, Vandevijvere S, Carriedo-Lutzenkirchen A (2020). Mechanisms for addressing and managing the influence of corporations on public health policy, research and practice: a scoping review. BMJ Open.

[11] The Convention Secretariat. Global progress in implementation of the WHO FCTC. 2021. Ninth session of the Conference of the Parties. https://untobaccocontrol.org/downloads/cop9/main-documents/FCTC_COP_9_5_EN.pdf. Accessed June 16, 2021.

[12] The Convention Secretariat. Examples of implementation of Article 5.3 communicated through the reports of the Parties. https://www.who.int/fctc/parties_experiences/en/.

[13] Hawkins B, Holden C (2018). European Union implementation of Article 53 of the Framework Convention on Tobacco Control. Global Health.

[14] United Nations Human Rights. Office of the High Commission. Guiding Principles for Business and Human Rights. 2011. https://www.ohchr.org/documents/publications/guidingprinciplesbusinesshr_en.pdf. Accessed June 14, 2021.

[15] United Nations Global Compact. Guidelines on a principle-based approach to the Cooperation between the United Nations and the business sector. https://www.unglobalcompact.org/library/3431. Accessed June 14, 2021.

